# Standardisation of lymphatic filariasis microfilaraemia prevalence estimates based on different diagnostic methods: a systematic review and meta-analysis

**DOI:** 10.1186/s13071-020-04144-9

**Published:** 2020-06-11

**Authors:** Natalie V. S. Vinkeles Melchers, Luc E. Coffeng, Sake J. de Vlas, Wilma A. Stolk

**Affiliations:** Department of Public Health, Erasmus MC, University Medical Center Rotterdam, P.O. Box 2040, 3000 CA Rotterdam, The Netherlands

**Keywords:** Lymphatic filariasis, Diagnostic comparison, Meta-regression, Counting chamber, Blood smear, Blood film, Knott’s technique, Membrane filtration

## Abstract

**Background:**

Lymphatic filariasis (LF) infection is generally diagnosed through parasitological identification of microfilariae (mf) in the blood. Although historically the most commonly used technique for counting mf is the thick blood smear based on 20 µl blood (TBS20), various other techniques and blood volumes have been applied. It is therefore a challenge to compare mf prevalence estimates from different LF-survey data. Our objective was to standardise microfilaraemia (mf) prevalence estimates to TBS20 as the reference diagnostic technique.

**Methods:**

We first performed a systematic review to identify studies reporting on comparative mf prevalence data as measured by more than one diagnostic test, including TBS20, on the same study population. Associations between mf prevalences based on different diagnostic techniques were quantified in terms of odds ratios (OR, with TBS20 blood as reference), using a meta-regression model.

**Results:**

We identified 606 articles matching our search strategy and included 14 in our analyses. The OR of the mf prevalences as measured by the more sensitive counting chamber technique (≥ 50 µl blood) was 2.90 (95% confidence interval (CI): 1.60–5.28). For membrane filtration (1 ml blood) the OR was 2.39 (95% CI: 1.62–3.53), Knott’s technique it was 1.54 (95% CI: 0.72–3.29), and for TBS in ≥ 40 µl blood it was 1.37 (95% CI: 0.81–2.30).

**Conclusions:**

We provided transformation factors to standardise mf prevalence estimates as detected by different diagnostic techniques to mf prevalence estimates as measured by TBS20. This will facilitate the use and comparison of more datasets in meta-analyses and geographic mapping initiatives across countries and over time.
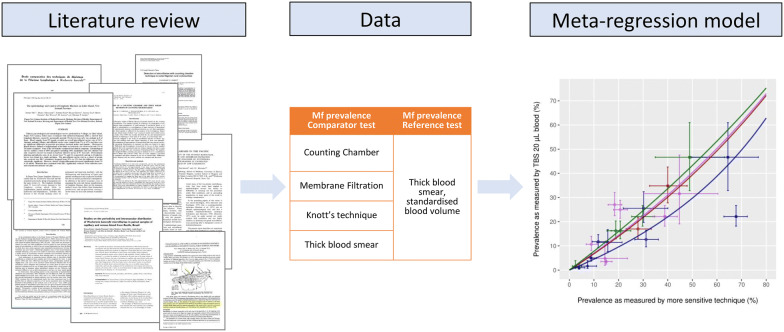

## Background

Lymphatic filariasis (LF) is a severely disabling, mosquito-borne infectious disease, causing elephantiasis and hydrocele. Active infection can be parasitologically diagnosed by demonstrating the presence of microfilaraemia (mf) in the blood, for which several methods are available. The diagnostic accuracy varies between methods, depending on timing of specimen collection (there is variation in the periodicity of LF parasite species in the blood), the type of blood sample (venous or capillary blood), blood volume examined, and blood sample processing methods (e.g. concentration, filtration, dehaemoglobinisation, etc.). The decision of the diagnostic techniques to be used in LF-surveys depends not only on the purpose of the survey and required diagnostic accuracy, but also on technical requirements, feasibility and acceptability to the surveyed population. Especially in remote rural areas, without sophisticated laboratory facilities, it is necessary to balance between a more sensitive *versus* practicable test when choosing techniques for detection of LF [1].

The accuracy of detecting mf is primarily influenced by the quantity of blood sampled [2], with larger blood volumes associated with higher sensitivity and higher mf prevalences. High diagnostic sensitivity is particularly important in settings with low mf densities, such as those close to elimination. Concentration methods with larger blood volumes may be more sensitive for the detection of especially low mf density infections, but collection of e.g. 1 ml venous blood volumes is not easily carried out in field conditions. Also, taking small capillary puncture samples may be more acceptable to the study population [3]. The assessment of geographical differences and trends over time in LF-survey results may be hindered by the use of different diagnostic techniques that each have different diagnostic sensitivities and specificities.

In an attempt to adjust detected mf prevalence estimates based on different diagnostic methods and blood volumes, Moraga et al. [4] identified studies reporting on paired estimates of LF prevalence based on 20 µl thick blood smear (TBS20) and membrane filtration technique (MFT) and concluded that the latter technique results in significantly higher mf prevalence estimates (*P* < 0.001). Moraga et al. [4] conclude insufficient quality data to derive adjustment terms for the standardisation of mf prevalence estimates for a range of transmission settings. Michael et al. [5] derived proportional transformation factors to translate observed mf prevalence as measured by examination of 20 or 100 µl blood into expected mf prevalences when using MFT of 1 ml blood, but such proportional transformation factors are less applicable in settings of relatively high initial mf prevalences. Sasa et al. [6] derived correction factors that were dependent on local endemicity levels for the standardisation of mf prevalence rates as detected by TBS with 30 µl from observed 10 and 20 µl blood volumes (0.616–0.961). None of these attempts systematically compared the most commonly used blood parasite detection techniques over a wide range of endemicity levels with the purpose to standardise mf prevalences to a reference technique.

Here, we performed a systematic review to identify studies reporting on paired mf prevalences as detected by various diagnostic techniques that can be used for the detection of LF blood parasites. From this empirical evidence, we developed a transformation model to standardise mf prevalences as measured by more sensitive diagnostic techniques to the historically most commonly used TBS20, and *vice versa*.

## Methods

### Data appraisal and extraction

We identified potentially relevant papers using a systematic search of the online databases Embase, Medline and PubMed, until 11th March 2019. The full search terms are described in Additional file 1: Text S1 (section 1). The title, abstract, full-text screening for eligibility of inclusion was based on whether the article would describe comparable mf prevalence estimates as measured by at least two different diagnostic methods, including TBS (regardless of the LF parasite species). The screening of titles and abstracts was performed independently by two authors (NVSVM, LEC). Inclusion of studies and study quality was then assessed by two authors (NVSVM, WAS). Studies were selected for data extraction if they were based on a representative population sample and if studies reported the number of individuals tested and the number of positive cases for both diagnostic techniques. We did not apply any language or publication date restrictions. Additional file 1: Text S1 (section 2) provides more detailed information on the data collection process. We have included a PRISMA checklist as Additional file 2: Table S2.

Data were extracted by one author (NVSVM) and entered into a standard Microsoft Excel template with full data entry validation by a second author (WAS). We extracted information on history of treatment prior to each survey, as well as the survey year, country, and specific location (e.g. district, village). We also extracted the number of positive cases or prevalence, as well as the total sampled population, blood volume, time of sampling, and species periodicity.

### Statistical analysis

Per study, associations between mf prevalence estimates based on different diagnostic techniques were quantified in terms of odds ratios (OR), with prevalence as measured by TBS20 as a reference, as this is historically the most commonly applied diagnostic tool [4]. TBS20 was defined as one blood slide containing one 20 µl blood spot (in some occasions we used 25 or 30 µl blood). ORs were then pooled between studies by means of meta-analysis and meta-regression models. To account for differences in sample size between studies, studies were weighted by the inverse of the variance of the log-OR, which was quantified using a normal approximation procedure (see Additional file 1: Text S1 (section 3)). We considered four model variants to describe the relation between the mf prevalence measured by TBS20 and the prevalence measured by another diagnostic technique: (i) a random-effects-only meta-analysis without any co-variates (i.e. “random-effects-only” model: the OR is not dependent on a diagnostic technique or observed mf prevalence); (ii) a mixed-effects meta-regression model with a coefficient for each diagnostic technique other than TBS20 (i.e. “intercept-only” model: the OR is allowed to vary between diagnostic techniques, but is not dependent on the observed mf prevalence); (iii) a mixed-effects meta-regression with a coefficient for the infection prevalence measured by TBS20 (i.e. “slope-only” model: the OR is allowed to vary with the observed mf prevalences, but is not dependent on the employed diagnostic technique); and (iv) a mixed-effects meta-regression with coefficients for each diagnostic technique and a coefficient for the infection prevalence measured by TBS20 (i.e. “slope-intercept” model: the OR is allowed to vary with the observed mf prevalences as well as between different diagnostic techniques to capture the potential association between diagnostic test sensitivity and the infection level in the population). Model variants were compared based on the corrected Akaike information criterion (AICc), a metric that converges to the AIC in case of large (infinite datasets) but in case of small datasets avoids favouring models with too many parameters [7]. All analyses were performed using the package *metaphor* [8] in R (version 3.5.1). A more detailed description of the methods, including mathematical formulae, is presented in Additional file 1: Text S1 (section 3). To be able to compare our results with earlier approaches for standardising mf prevalences based on scaling factors, we translated the model-predicted ORs to relative risk (RR) functions; because for a given OR, the RR depends on the prevalence of the reference group, we report derived RRs for different levels of prevalences based on TBS20. Uncertainty of standardisation of mf prevalence to TBS20 based on the intercept-only model was quantified analytically by directly propagating parameter uncertainty as estimated by the meta-regression model, whereas a Monte Carlo approach was used for the slope-only model (Additional file 1: Text S1 (section 3)).

## Results

**Fig. 1:**
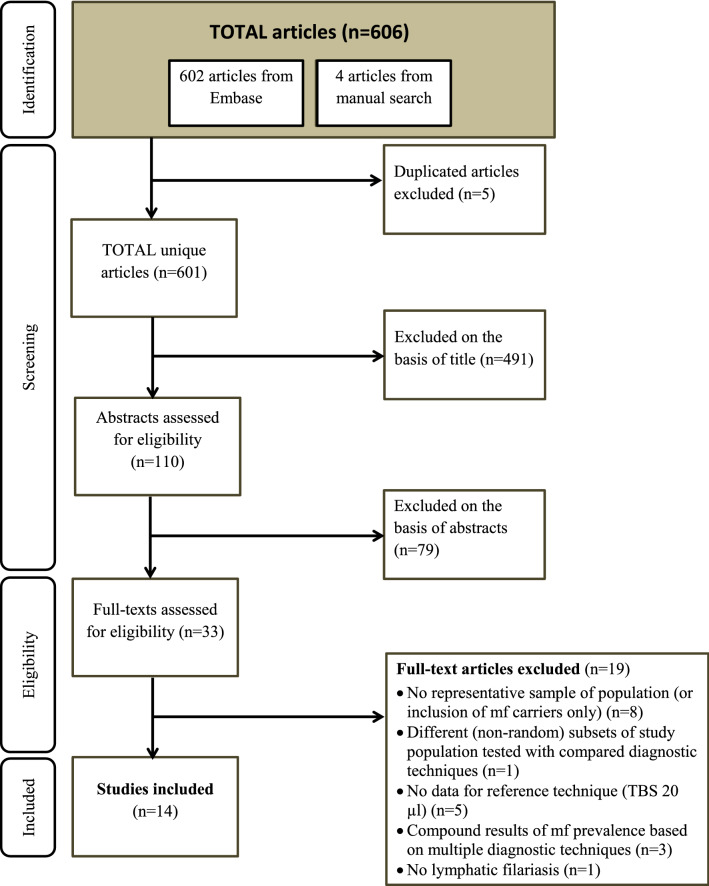
Flow chart of search strategy

**Fig. 2:**
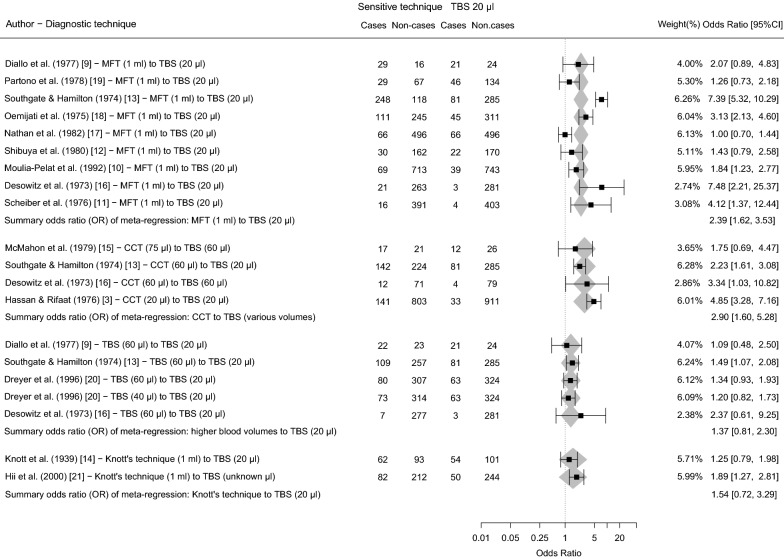
Forest plot of all included studies with LF mf cases and non-cases as measured by the more sensitive diagnostic technique *versus* TBS20 blood, presented per diagnostic technique. The observed study-level ORs (black squares, and OR-column) with 95% confidence intervals, the weight of each record, and ORs as predicted by the intercept-only model (grey diamonds) are shown

We identified in total 602 unique articles, and included 14 full-text articles based on the inclusion- and exclusion criteria. The flow chart of the search strategy is presented in Fig. 1. The included studies were published between 1939 and 2000. Seven studies did not explicitly report whether the sampled population had received mass treatment or not [3, 9–14], although we could often tentatively conclude from study year and location that no large-scale control activities had taken place. In one study, blood sampling was done during day-time after provocation with a single 100 mg dose of diethylcarbamazine citrate (DEC) [15]. Surveys from included studies were performed across 13 LF-endemic countries (see Additional file 1: Table S1). Most commonly, mf prevalences were compared using MFT and TBS20 (nine studies) [9–13, 16–19]. Four studies diagnostically compared mf prevalences as measured by various blood volumes using the counting chamber technique (CCT) with various blood volumes of TBS [3, 13, 15, 16]. We identified five records from four studies reporting comparative mf prevalences between TBS20 *versus* TBS with higher blood volumes (≥ 40 µl blood) [9, 13, 16, 20]. Only two included studies reported on the comparative mf prevalence between Knott’s technique and TBS20 [14, 21]. In several occasions (Fiji [13, 16], Brazil [20], Tanzania [15]), blood sampling was based on day-time sampling, even though in Brazil and Tanzania the LF parasite species are known to have a nocturnal periodicity. The study-level ORs as well as the pooled ORs of each diagnostic comparison are presented in Fig. 2.

The AICc of the random-effects-only model was 40.3, but this reference model did not provide insight into differences between diagnostic tests and was therefore not used in our further analysis of conversion factors. Comparing the three meta-regression model variants, we found that the slope-only model was the most parsimonious model with the lowest AICc (AICc: 39.6). The AICc of the intercept-only model and the slope-intercept model were 44.5 and 48.7, respectively. However, the slope-only model resulted in an epidemiologically implausible pattern where the OR for detection of mf by the more sensitive diagnostic test (compared to TBS20) would eventually become smaller than 1.0 for very high mf prevalences. The shape of the association deviated too much from the data points above an observed mf prevalence of 50% as measured by a more sensitive technique (see Additional file 1: Figures S1, S2). Results and model outputs presented below are therefore based on the intercept-only model.

**Fig. 3:**
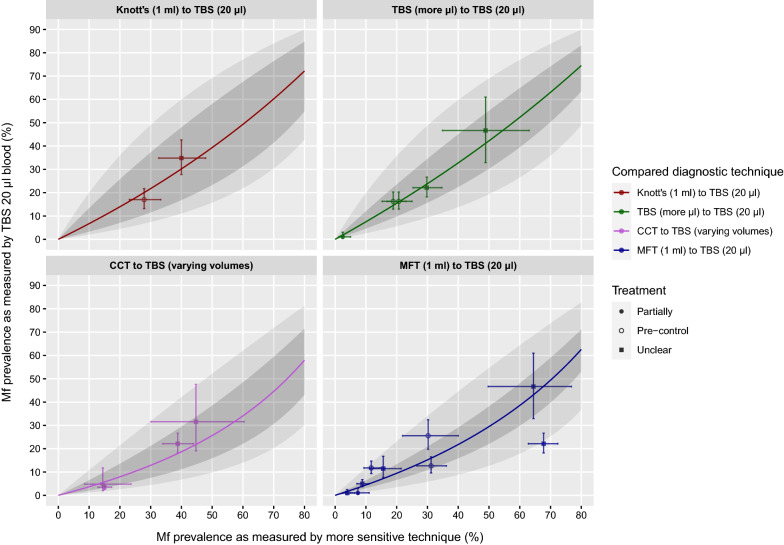
Model-predicted mf prevalences as measured by TBS20, given an mf prevalence based on a more sensitive diagnostic technique (horizontal axis, panels for different techniques). Dark grey bands represent 95%-confidence intervals for uncertainty of the population mean; light grey bands represent prediction uncertainty. Bullets represent data used to train the model, with horizontal and vertical error bars representing 95% confidence intervals for binomial sampling error. Different shapes of the dots characterise data from pre-control settings, data from settings where only some villages were treated (partially), or data from settings where it is unclear whether treatment has been provided

**Fig. 4:**
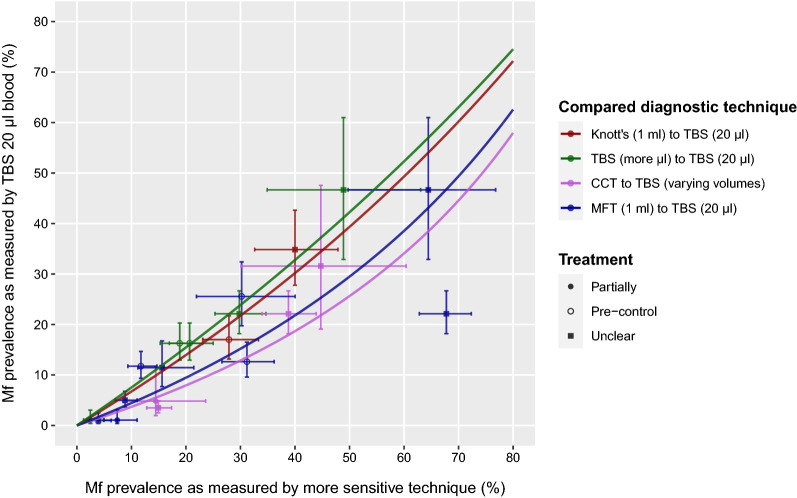
The combined results of the predicted association of mf prevalences as measured by a more sensitive diagnostic technique and TBS20 against the observed data with horizontal and vertical 95% CI around the data. Different diagnostic techniques are represented by the different coloured lines. Different shapes of the dots characterise data from pre-control settings, data from settings where only some villages were treated (partially), or data from settings where it is unclear whether treatment has been provided. Predictions lines on the right denote a higher diagnostic sensitivity

According to the intercept-only model, TBS20 has a lower sensitivity for detecting the presence of mf than the other tests in the comparisons, as indicated by the ORs above 1.0 (Fig. 2). For both CCT and MFT, the ORs were significantly different from 1.0 (*P* = 0.0005 and *P* < 0.0001, respectively). CCT was the most sensitive (highest OR), followed by MFT, Knott’s technique, and lastly TBS in ≥ 40 µl/blood (lowest OR). Figure 3 illustrates the model-predicted association between mf prevalences as detected by TBS20 and each of the more sensitive techniques. The model-predicted mf prevalences by TBS20 increased non-linearly, and followed the observed data adequately. Treatment history does not seem to influence any of the associations. Figure 4 shows the performance of the different diagnostic techniques for the detection of mf and their mutual relationship.

To facilitate future translation of mf prevalences as detected by CCT, MFT, Knott’s technique and TBS with higher blood volumes to TBS20, Additional file 3: Data S1 holds the data underlying the curves and confidence bands from Fig. 3. Clearly, the transformation factors for each diagnostic technique depend on endemicity level. For example, the RR for detection of mf with TBS20 as compared to the more sensitive CCT was estimated at 0.41 (95% CI: 0.24–0.69) at 10% mf prevalence and 0.61 (95% CI: 0.41–0.83) at 40% mf prevalence (as measured by TBS20).

## Discussion

Blood parasitology for detection of active LF infection has traditionally been the preferred method, yet there is no gold standard for the diagnostic technique that should be applied. The choice of the diagnostic technique for detection of blood mf depends on several factors, e.g. geographical region, implementation setting, and availability of laboratory equipment. We have performed a systematic review and meta-analyses in order to standardise mf prevalences as measured by a range of diagnostic techniques for blood parasite detection to mf prevalences as measured by the historically most commonly used TBS20. Standardisation of LF blood parasite prevalences using various diagnostic techniques assists a wide group of end-users (e.g. researchers, policy-makers, drug developers) to compare LF-survey data that were collected using different blood volumes or detection techniques. Especially cross-regional comparison of LF data is now facilitated, as certain diagnostic tools are more frequently used in certain LF-endemic regions across the globe, i.e. the use of CCT in predominantly Africa and MFT in the Western Pacific.

Multiple meta-regression models were compared with each other, and the intercept-only model was selected, even though the slope-only model was statistically the most parsimonious model. The predicted regression line as obtained by the slope-only model, however, led to an epidemiologically implausible pattern, even though it fitted the data best. This was likely due to the unequal weighting of some data points with wide 95% confidence intervals above 50% mf prevalence as measured by the more sensitive techniques. We found that the sensitivity of CCT was higher than MFT and Knott’s technique, which can be explained by the fact that mf intensity is higher in finger prick blood than in venous blood. For this reason, 60–100 µl blood by finger prick (CCT) would be more sensitive than 1 ml blood (MFT or Knott’s technique).

One included study mentioned co-endemicity of *Mansonella* species in the blood samples that were tested [17]. As this paper reported their results for *Wuchereria bancrofti* and *M. ozzardi* separately, this does not introduce a bias in our analysis. Considering the occurrence of *Mansonella* species in regions of Central Africa [22], and Central and South America [23], co-endemicity might play a role in four other studies [9, 11, 15, 20], although this was not examined. Unrecognised co-endemicity could lead to overestimation of the LF mf prevalence. Literature reviews on the distribution of *Mansonella* species report a wide variation in community prevalence and an estimated average prevalence of about 11–23% in the population across *Mansonella*-endemic areas [22–24]. We expect that *Mansonella* infection affects all diagnostic techniques to a similar extent and therefore has limited impact on the derived associations.

There is variation between studies in setting characteristics, study population composition, and diagnostic procedures, as described in Additional file 1: Table S1. For example, there were two studies with three data points [15, 20] that performed day-time sampling although the parasite species had nocturnal periodicity. If we would omit these three data points (two data points for TBS with more microliters [20] and one data point for CCT [15]), this would barely influence the predicted association (the association for TBS with more microliters would not change at all; the associating for CCT would become slightly more sensitive than the current derived association in Fig. 4). Similarly, if we would exclude studies that used ear prick or venepuncture for the TBS20 blood spot rather than finger prick, the overall pattern of the association between the four different diagnostic techniques somewhat changed (MFT would become slightly more sensitive, comparable with CCT). Finally, there were several studies [3, 9–14, 20] for which it was unclear whether mass treatment had occurred among the study population or not. Considering the old publication dates, the likelihood that mass treatment among the population would have occurred seems small. Figures 3 and 4 do not show any other association between the diagnostic techniques for studies with different treatment history.

Some previous attempts were made by others to standardise mf prevalences as measured by one diagnostic technique for blood parasite detection to another [4, 5]. Our model predictions are an improvement over previous methods by using ORs through a logit link, limiting the predicted mf prevalence by TBS µl blood between 0 and 1, for each given observed mf prevalence as measured by a more sensitive diagnostic technique. Due to a different methodological approach to standardise mf prevalences to TBS20 than previously used by others [4–6], it is challenging to directly compare our study results. Michael et al. [5] reported a fixed factor of 1.95 for transformation of mf prevalence estimates based on examination of TBS20 to that based on 1 ml MFT. The inverse of this factor (thus from MFT to TBS20) is 0.51, which is within the range of 0.48 to 0.65 as found in our analysis for a 10% to 40% mf prevalence as measured by TBS20, respectively. A correction factor should clearly not be a constant measure over different epidemiological settings but should be allowed to vary over different community-based prevalences. This is in line with the work by Sasa et al. [6] who also reported varying transformation factors that were dependent on local transmission settings.

## Conclusions

Our results facilitate the comparison of mf prevalence data that are collected historically or at present, with blood mf detected through various diagnostic techniques in areas of different transmission intensities. These results are useful for e.g. meta-analyses and geographical mapping initiatives. They can thus assist LF programme managers, policy-makers, and researchers by evaluating the progress made during mass drug administration programmes towards the LF elimination targets set by the World Health Organization. These results thus enable the end-users to evaluate potential changes in mf prevalence over time and by geographical area, while using different diagnostic techniques and inherent blood volumes for LF blood parasite detection.

## Supplementary information

**Additional file 1: Text S1.** Detailed methods and additional results. **Table S1.** Summary table with main characteristics of included studies reporting on comparative mf prevalences as measured by thick blood smears versus more sensitive diagnostic techniques. **Figure S1.** Odds ratio of microfilariaemia as measured by the more sensitive diagnostic technique *versus* TBS with 20 μl blood, plotted against the prevalence of TBS20. **Figure S2.** Model-predicted mf prevalences as measured by TBS 20 μl blood, given a mf prevalence based on a more sensitive diagnostic technique.

**Additional file 2: Table S2.** Preferred reporting items for systematic reviews and meta-analyses (PRISMA) checklist.

**Additional file 3: Data S1.** Predicted prevalence estimates for the various diagnostic techniques, with 95% confidence intervals, and relative risks, for transformation to mf prevalences as measured by TBS20 and *vice versa*.

## Data Availability

All data generated or analysed during this study are included in this published article and its additional files.

## Abbreviations

AICc: corrected Akaike information criterion
CCT: counting chamber technique
95% CI: 95% confidence interval
DEC: diethylcarbamazine citrate
LF: lymphatic filariasis
mf: microfilariaemia
MFT: membrane filtration technique
OR: odds ratios
RR: relative risk
TBS20: thick blood smear based on 20 µl blood
