# Supplementary material for: Standardisation of lymphatic filariasis microfilaraemia prevalence estimates based on different diagnostic methods: a systematic review and meta-analysis
Source: Parasit Vectors. 2020 Jun 11;13:302. doi: 10.1186/s13071-020-04144-9 (PMC7288683; doi:10.1186/s13071-020-04144-9)
Supplement: Supplementary file 1 — Additional file 1: Text S1. Detailed methods and additional results. Table S1. Summary table with main characteristics of included studies reporting on comparative mf prevalences as measured by thick blood smears versus more sensitive diagnostic techniques. Figure S1. Odds ratio of microfilariaemia as measured by the more sensitive diagnostic technique versus TBS with 20 μl blood, plotted against the prevalence of TBS20. Figure S2. Model-predicted mf prevalences as measured by TBS 20 μl blood, given a mf prevalence based on a more sensitive diagnostic technique. [file 13071_2020_4144_MOESM1_ESM.pdf]

Additional File1

Detailed methods and additional results

Supplement to:

Standardisation of lymphatic filariasis microfilaraemia prevalence estimates based on different diagnostic methods: a systematic review and meta-analysis

Natalie V.S. Vinkeles Melchers[1#], Luc E. Coffeng[1], Sake J. de Vlas[1], Wilma A. Stolk[1*]

[1] Department of Public Health, Erasmus MC, University Medical Center Rotterdam, P.O. Box 2040, 3000 CA Rotterdam, The Netherlands.

# Correspondence to:
Natalie VS Vinkeles Melchers, Department of Public Health, Erasmus MC, University Medical Center Rotterdam, P.O. Box 2040, 3000 CA Rotterdam, The Netherlands, n.vinkelesmelchers@erasmusmc.nl, Natalie.melchers@gmail.com; +31 (0)10 70 38465

* Alternative:
Wilma A Stolk, Department of Public Health, Erasmus MC, University Medical Center Rotterdam, P.O. Box 2040, 3000 CA Rotterdam, The Netherlands, w.stolk@erasmusmc.nl.

Table of Contents

1. Search terms 3
2. Data collection process 4
3. Details of statistical analysis 5
Data preparation 5
Quantification of uncertainty in standardised mf prevalence 5
4. Additional results 7
References 13

1. Search terms

We applied the following search terms (box 1) in the online database Embase, including Medline and PubMed. The search included published articles up to 11th of March 2019. No restrictions in the year of publication or language were applied. Conference abstracts, reviews, letters and editorial notes were excluded from the search. Manual search of other relevant articles was performed by one of the authors (NVSVM) through cross-checking the references of the included studies.

Box 1: Search terms used to identify relevant articles in Embase.

**(**'lymphatic filariasis'/exp OR 'Wuchereria'/exp OR 'Brugia'/exp *OR 'microfilariasis'/de* OR (bancrofti* OR Wuchereria* OR brugia OR brugian *OR microfilariasis OR microfilaremia* OR microfilaraem* OR ((microfilaria) NEAR/3 (positiv* OR negativ* OR blood OR serum))*):ab,ti**) AND (**'diagnostic test'/de/**mj** OR 'blood filtration'/de OR 'capillary blood'/de OR 'venous blood'/de OR 'blood smear'/de OR 'blood film'/de *OR 'blood smear'/de* OR 'membrane filter'/de OR (filtrat* OR ((capillary* OR venous) NEAR/3 (blood)) *OR ((blood OR thick) NEAR/3 (smear* OR film))* OR ((count*) NEAR/3 (chamber* OR microfilaria*)) OR ((membrane*) NEAR/3 (filter*)) OR Knott*):ab,ti**) NOT** ([Conference Abstract]/lim OR [Letter]/lim OR [Note]/lim OR [Editorial]/lim)

2. Data collection process

We identified 602 articles to be screened and found four more references through manual search (see **Figure 1)**. Out of the 606 published articles identified, five duplicates were excluded. A three-step approach was used for the screening of relevant full-text articles performed by two reviewers (NVSVM and LEC), i.e. title screening, abstract screening of included titles, and full-text screening of included abstracts. During this process, we excluded title and abstract if the text suggested that data on mf prevalences as measured by multiple blood parasite diagnostic techniques, including at least the thick blood smear (TBS), was not present.

All eligible full-text articles for inclusion were assessed by two authors (NVSVM, WAS) for final inclusion in the study. Studies were included if they reported community-based mf prevalence, or number of positive cases, and total study size, detected by at least two different diagnostic tests, including at least TBS based on 20 µL blood (TBS20), sampled at the same time point. Compound results of mf prevalence or number of cases based on multiple diagnostic techniques were not used. Studies were excluded if they failed to report sample sizes (i.e. either number of positive and negative cases or a denominator combined with prevalence). Studies were also excluded if they did not include a representative sampling of the population of interest (e.g. villages, districts, immigrant groups, or schools), such as mf carriers, individuals with LF symptoms, household members of mf carriers. If a non-random subset of the study population was used for the various diagnostic techniques, the study would also be excluded. Articles reporting results with animals as study population were evidently also excluded. Assessment of potential study-specific risk of bias was performed by two authors (NVSVM, WAS), and is described in **Table S1**.

After selection of full-texts, data extraction was performed. We extracted the mf prevalence or number of positive and negative cases, as well as the total sampled population, diagnostic technique, blood volume of each diagnostic technique, time of sampling, and parasite periodicity from each study. In the few occasions that surveys also reported on age- and sex-stratified mf prevalence measured separately by ≥2 diagnostic tools, we extracted the data. However, too little data was obtained to derive appropriate sex- and age-stratified transformation factors. Data extraction was performed by one author (NVSVM) and cross-validated by a second author (WAS). Discordance in data entry was resolved by consensus.

3. Details of statistical analysis

In this section, we present the details of the statistical analysis of the association between mf prevalences as measured by TBS20 and four more sensitive diagnostic techniques.

Data preparation

To perform the random-effects-only meta-analysis and/or the three meta-regressions, studies were summarised in terms of odds ratios (OR) for detection of microfilariae by the more sensitive diagnostic technique compared to TBS20. To reduce heteroscedasticity, ORs were log-transformed and uncertainty in log-ORs was quantified based on the following normal approximation:

$$\text{var}(\text{log-OR}_i) = \frac{1}{a_i} + \frac{1}{b_i} + \frac{1}{c_i} + \frac{1}{d_i}$$

Here, a_i, b_i, c_i, and d_i represent the number of positive (a_i, c_i) and negative cases (b_i, d_i) for the two diagnostic techniques being compared. To account for differences in sample size between studies, studies weighted by the inverse of the variance of the log-OR.

Quantification of uncertainty in standardised mf prevalence

Uncertainty of standardisation of mf prevalence to TBS20 based on the intercept-only model was quantified analytically by directly propagating parameter uncertainty as estimated by the meta-regression model. We also quantified the uncertainty in mf prevalence standardisations to TBS20 based on the slope-only model. To do so, a Monte Carlo approach was taken as there is no way to rearrange the regression formula into a closed-form expression for the prediction of mf prevalences based on TBS20 through which uncertainty could be analytically propagated. This was because mf prevalences as measured by TBS20 turns up on both sides of the equation, with one instance occurring within the logit function and another outside:

$$p_{TBS20} = \frac{1}{1 + e^{\left(-\left[\log\left(\frac{\text{p}_{\text{other test}}}{1-\text{p}_{\text{other test}}}\right)+\beta_o+\beta_1 \cdot p_{TBS20}\right]\right)}}$$

We drew 10,000 samples of model parameter values from a multivariate normal distribution with mean equal to the estimated model coefficients and variance equal to the variance-covariance matrix of model coefficients. For each sample of parameter values, we then standardised mf prevalence to TBS20 by numerically solving the equation with a Broyden–Fletcher–Goldfarb–Shanno (BFGS) algorithm provided within the *optim* function in R (version 3.5.1). Last, we quantified uncertainty as 95%-confidence intervals, which were calculated as the 2.5th and 97.5th percentile of the standardised prevalences resulting from the Monte Carlo simulations.

4. Additional results

Table S1. Summary table with main characteristics of included studies reporting on comparative mf prevalences as measured by thick blood smears versus more sensitive diagnostic techniques. *Abbreviation*: NA, Not available.

Author and year of publication [ref]	Year of survey	Country of survey	Community surveyed (total N sampled)	Pre-control	Periodicity of parasite	Timing of sampling	Mf prevalence by TBS 20µL blood	Blood volume of second diagnostic test	Mf prevalence as measured by second test	Potential sources of bias
**Higher volume thick blood smears**										
Desowitz *et al.* 1973 [1]	1971	Fiji	Several villages Viti Levu (N=284)	Partially	Sub-periodic or aperiodic	Day	1·7%	60 µL	2·5%	One village (Bivienua) did receive DEC since 1953, other villages never received MDA. Population sampling method unclear.
Diallo *et al.* 1977 [2]	NA	Senegal	Bousnack Ngourbane (N=45)	NA	Nocturnal	Night	46·7%	60 µL	48·9%	Voluntary participation to study. Includes only individuals between 15-60 years of age.
Dreyer *et al.* 1996 [3]	NA	Brazil	Greater Recife (N=774)	NA	Nocturnal	Day	16·3%	60 µL	20·7%	Unclear if volunteering sample population were patients from the Filariasis clinic, or if they were residences that volunteered blood sampling at the clinic. TBS20 based on 60 mm fingerprick (60 mm of blood collected, and expelled over three strips of 20 µL blood smear each for TBS).
								40 µL	18·9%	
Southgate & Hamilton 1974 [4]	1971	Fiji	2 villages in Viti Levu (N=366)	NA	Sub-periodic or aperiodic	Day	22·1%	60 µL	29·8%	Many young males were absent during survey.
**Membrane filtration technique**										
Desowitz *et al.* 1973 [1]	1971	Fiji	Several villages Viti Levu	Partially	Sub-periodic or aperiodic	Day	1·7%	1 mL	7·4%	One village (Bivienua) did receive DEC since 1953, other villages never received MDA. Population sampling

			(N=284)							method unclear.
Diallo *et al.* 1977 [2]	NA	Senegal	Bousnack Ngourbane (N=45)	NA	Nocturnal	Night	46·7%	1 mL	64·4%	Voluntarily participation to study. Includes only individuals between 15-60 years of age.
Moulia-Pelat *et al.* 1992 [5]	NA	French Polynesia	Moorea village, Society island (N=782)	NA	Sub-periodic or aperiodic	NA	5.0%	1 mL	8.8%	TBS20 based on 40 mm fingerprick (40 mm of blood collected, and expelled over two strips of 20 µL blood smear each for TBS).
Nathan 1982 [6]	1978-1979	Trinidad	Blanchisseuse (N=564)	Pre-control	Nocturnal	Night	11·7%	1 mL	11·7%	No indication that treatment was ever provided in this area, so almost certainly pre-control. Blood sample for TBS through earprick rather than fingerprick. *Mansonella spp.* co-endemic, but results were presented separately, so here we only report *W. bancrofti* results.
Oemijati *et al.* 1975 [7]	NA	Indonesia	Kepu district (N=356)	Pre-control	Nocturnal	Night	12·6%	1 mL	31·2%	No direct results available of mf prevalence by TBS20, but assumed that remaining % of total infections of low-density (MFT-detectable only) were also detected by TBS20.
Partono *et al.* 1978 [8]	1977	Indonesia	Karakuak village (N=200)	Pre-control	Nocturnal	Night	25·6%	1 mL	30·2%	Newly developed village (max. 10 years existence) with part from population originally from non-endemic areas. We included TBS20 results of 180 individuals (we excluded 20 children of <5 years of age); a randomly selected subset of the study population was also examined by MFT of 1-2 mL venous blood collection. *Brugia timori* parasite species.
Scheiber *et al.* 1976 [9]	1975	Togo	4 villages near Mono river (N=407)	NA	Nocturnal	Night	1·0%	1 mL	3·9%	Study performed in villages where seven years earlier a much higher mf prevalence was found [10]. TBS20 based on venous blood (1.1 ml of blood collected through venepuncture, of which 0.02 ml was

										used for TBS).
Shibuya *et al.* 1980 [11]	NA	Philippines	Irosin, Sorsogon (N=483)	NA	Nocturnal	Night	11·5%	1 mL	15·6%	Blood sample was collected through 2.2 ml venepuncture, and 0.2 ml used for 30µL blood smear.
Southgate & Hamilton 1974 [4]	1971	Fiji	2 villages in Viti Levu (N=366)	NA	Sub-periodic or aperiodic	Day	22·1%	1 mL	67·8%	Many young males were absent during survey. Likely pre-control, but no specified information.
	**Knott's technique**									
Hii *et al.* 2000 [12]	1993	Papua New Guinea	9 villages on Lihir Island (N=593)	Pre-control	Nocturnal	Night	17·0%	1 mL	27·9%	-
Knott 1939 [13]	NA	NA	Labour camp on Virgin Islands (?) (N=155)	NA	Nocturnal	Night	34·8%	1 mL	40·0%	Study performed among labour camp population of boys 16-22 years. TBS20 estimated on assumption that TBS20 would be negative among individuals without mf in 10 c.c. blood.
	**Counting Chamber Technique***									
Desowitz *et al.* 1973 [1]	1971	Fiji	Several villages Viti Levu (N=284)	Partially	Sub-periodic or aperiodic	Day	4·8%	60 µL	14·5%	One village (Bivienua) did receive DEC since 1953, other villages never received MDA. Population sampling method unclear.
Hassan & Rifaat 1976 [14]	NA	Egypt	Arab-Ebs, Kalubia (N=944)	NA	Nocturnal	Night	3·5%	20 µL	14·9%	Low blood volume for CCT (20 µL/blood).
McMahon 1979 [15]	NA	Tanzania	NA	Possibly	Nocturnal	Day	31·6%	75 µL	44·7%	Day sampling was performed using provocative sampling using a single dose (100mg) of diethylcarbamazine citrate (DEC). Absence of information on study sample (selection) or study area. Potential mass treatment with DEC in the region [16]. TBS20 based on 60 mm fingerprick (60 mm of blood collected, and expelled over

										three strips of 20 µL blood smear each for TBS).
Southgate & Hamilton 1974 [4]	1971	Fiji	2 villages in Viti Levu (N=366)	NA	Sub-periodic or aperiodic	Day	X	60 µL	38·8%	Many young males were absent during survey. Likely pre-control, but no specified information.

Note:
* Due to limited reported comparative mf prevalences by parallel diagnostic tools with TBS (20 µL) and CCT, we included studies that compared the CCT (any volume) to the TBS with any blood volume rather than 20 µL blood volumes.

Figure S1. Odds ratio (OR, y-axis) of microfilaraemia (bullets) as measured by the more sensitive diagnostic technique (colours) versus TBS with 20 µL blood, plotted against the prevalence of TBS20 (x-axis). Vertical lines represent 95% confidence intervals of the ORs. Solid coloured lines represents the OR as estimated by the intercept-only meta-regression model (intercept for each diagnostic technique and no dependence on TBS20 prevalence). The dashed black line represents the OR as predicted by the slope-only meta-regression model, which assumes that 1) the log-OR is linearly associated with mf prevalence as measured by TBS20, and 2) there is no systematic difference between the four more sensitive diagnostic techniques (one shared intercept). Different shapes of the dots characterise data from pre-control settings, data from settings where only some villages were treated (partially), or data from settings where it is unclear whether treatment has been provided.

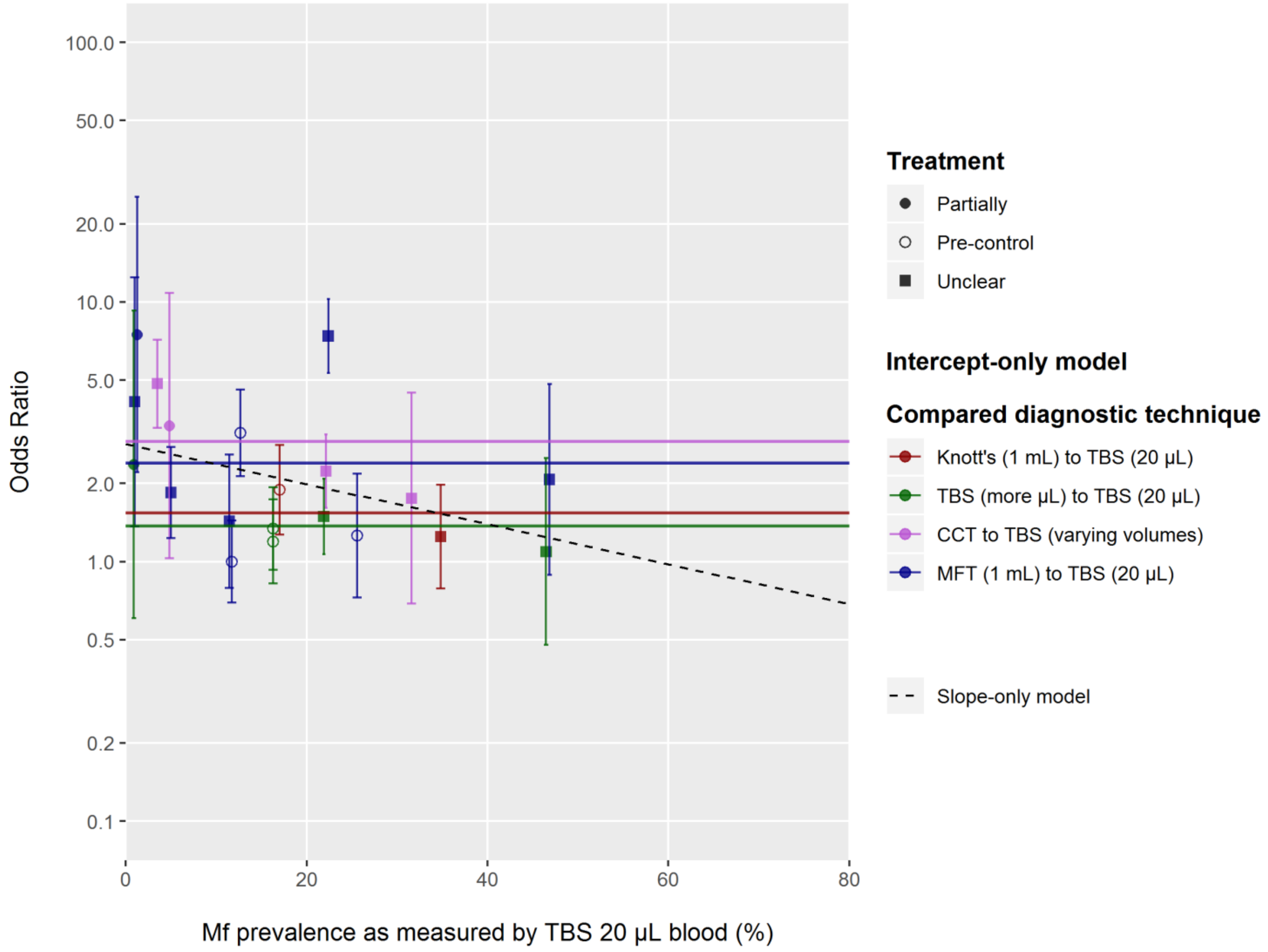

Figure S2. Model-predicted mf prevalences as measured by TBS 20 μL blood (vertical axis), given a mf prevalence based on a more sensitive diagnostic technique (horizontal axis). Bullets represent data used to train the models, with horizontal and vertical error bars representing 95%-confidence intervals for binomial sampling error. Different colours represent the different (more sensitive) diagnostic techniques. Solid coloured lines represent the association as predicted by the intercept-only meta-regression model (intercept for each diagnostic technique and no dependence on TBS20 prevalence). The dashed black line represents the association as predicted by the slope-only meta-regression model, which assumes that 1) the log-OR is linearly associated with mf prevalence as measured by TBS20, and 2) there is no systematic difference between the four more sensitive diagnostic techniques (one-shared intercept). Different shapes of the dots characterise data from pre-control settings, data from settings where only some villages were treated (partially), or data from settings where it is unclear whether treatment has been provided.

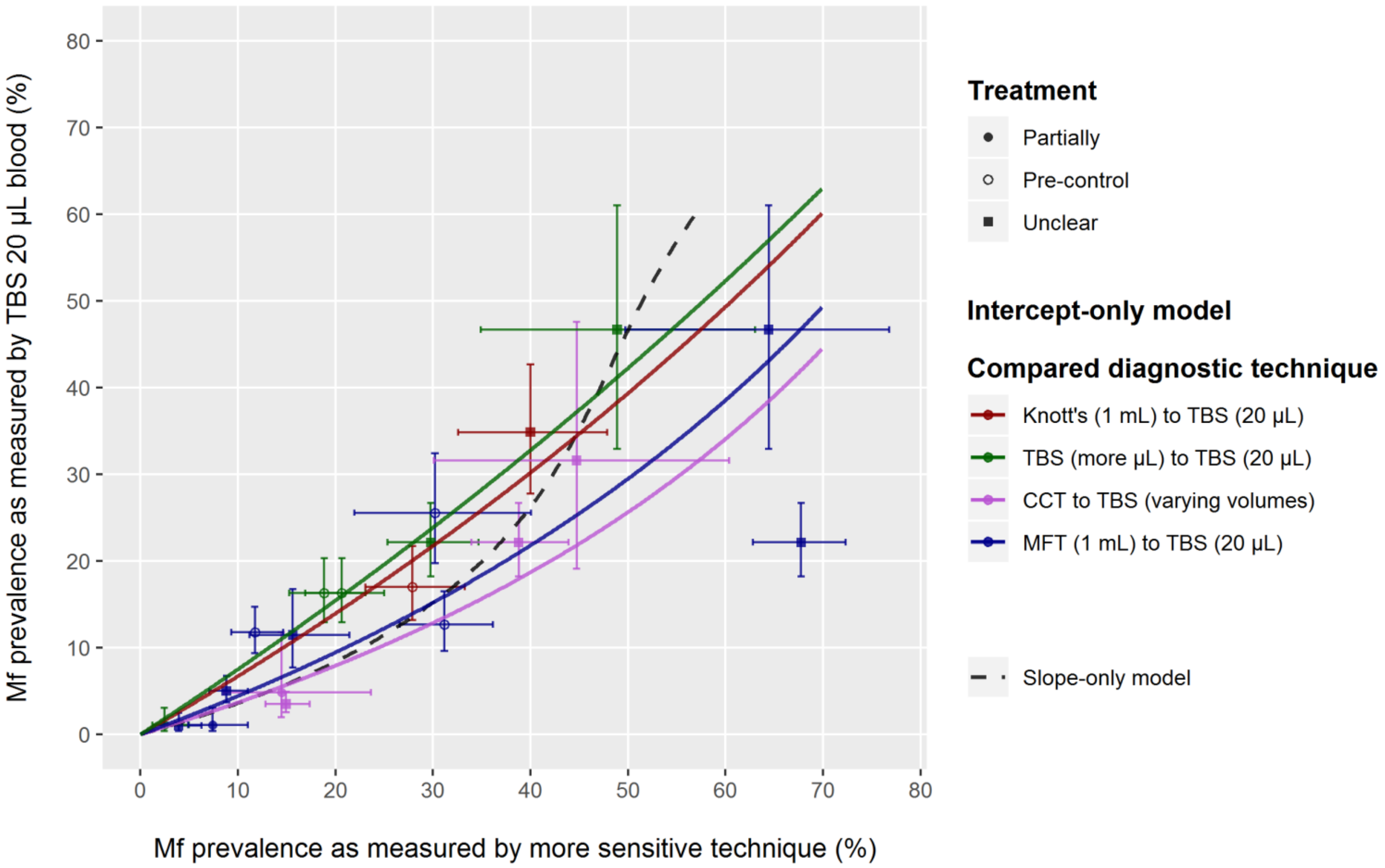

References

1. Desowitz RS, Southgate BA, Mataika JU. Studies on filariasis in the Pacific 3. Comparative efficacy of the stained blood film, counting chamber and membrane filtration techniques for the diagnosis of *Wuchereria bancrofti* microfilaraemia in untreated patients in areas of low endemicity. Southeast Asian J Trop Med Public Health. 1973;4:329–35.

2. Diallo S, Sarr M, Coulibaly A, Diouf M, Diagne S. Etude comparative des techniques de dépistage de la Filariose lymphatique à *Wuchereria bancrofti*. Bull Soc Med Afr Noire Lang Fr. 1977;22:146–53.

3. Dreyer G, Pimentael A, Medeiros Z, Béliz F, Moura I, Coutinho A, et al. Studies on the periodicity and intravascular distribution of *Wuchereria bancrofti* microfilariae in paired samples of capillary and venous blood from Recife, Brazil. Trop Med Int Heal. 1996;1:264–72.

4. Southgate BA, Hamilton PJS. A quantitative approach to parasitological techniques in bancroftian filariasis and its effect on epidemiological understanding. Trans R Soc Trop Med Hyg. 1974;68:177–86.

5. Moulia-Pelat JP, Glaziou P, Nguyen-Ngoc L, Cardines D, Cartel JL. A comparative study of detection methods for evaluation of microfilaremia in lymphatic filariasis control programmes. Trop Med Parasitol. 1992;43:146–8.

6. Nathan MB, Beckles G, Tikasingh ES, Hamilton PJ, Monteil S. Parasitological and clinical studies of *Wuchereria bancrofti* and *Mansonella ozzardi* in coastal north Trinidad, West Indies. West Indian Med J. 1982;31:168–76.

7. Oemijati S, Desowitz RS, Partono F, Pant CP, Mechfudin H, Sajidiman H. Studies on filariasis in the Pacific. 4. The application of the membrane filter concentration technique to a survey of *Wuchereria bancrofti* filariasis in Kepu district, Jakarta, Indonesia. Southeast Asian J Trop Med Public Health. 1975;6:186–9.

8. Partono F, Pribadi PW, Soewarta A. Epidemiological and clinical features of *Brugia timori* in a newly established village, Karakuak, West Flores, Indonesia. Am J Trop Med Hyg. 1978;27:910–5.

9. Scheiber P, Braun-Munzinger RA, Southgate BA. Bancroftian Filariasis in Togo 1. A comparative field study of the membrane filtration concentration technique and conventional blood films. Tropenmed Parasitol. 1976;27:224–8.

10. Brengues J, Subra R, Bouchité B. Etude parasitologique, clinique et entomologique sur la filariose de Bancroft dans le Sud du Dahomey et du Togo. Médicale Parasitol. 1969.

11. Shibuya T, Cabrera BD, Tanaka H. Comparison of the blood film, millipore filter and nuclepore filter techniques for the detection of microfilaremia in a field survey in the Philippines. Jpn J Exp Med. 1980;50:463–8.

12. Hii J, Bockarie MJ, Flew S, Genton B, Tali A, Dagoro H, et al. The epidemiology and control of lymphatic filariasis on Lihir Island, New Ireland Province. P N G Med J. 2000;43:188–95.

13. Knott J. A method for making microfilarial surveys on day blood. Trans R Soc Trop Med Hyg. 1939;33:191–6.

14. Hassan Z, Rifaat MA. Comparative efficacy of the stained blood-film and counting-chamber technique for the diagnosis of *Wuchereria bancrofti*. J Egypt Public Health Assoc. 1976;51:223–8.

15. McMahon JE, de Marshall TFC, Vaughan JP, Abaru DE. Bancroftian filariasis: A comparison of microfilariae counting techniques using counting chamber, standard slide and membrane (nuclepore) filtration. Ann Trop Med Parasitol. 1979;73:457–64.

16. McMahon JE, Marshall TF, Vaughan JP, Kolstrup N. Tanzania Filariasis Project: a provocative day test with diethylcarbamazine for the detection of microfilariae of nocturnally periodic *Wuchereria bancrofti* in the blood. Bull. World Health Organ. 1979.
